# Gene-Based Antibody Strategies for Prion Diseases

**DOI:** 10.1155/2013/710406

**Published:** 2013-08-21

**Authors:** Alessio Cardinale, Silvia Biocca

**Affiliations:** ^1^IRCCS San Raffaele Pisana, Via di Val Cannuta 247, 00166 Rome, Italy; ^2^Department of Systems Medicine, University of Rome “Tor Vergata,” Via Montpellier 1, 00133 Rome, Italy

## Abstract

Prion diseases or transmissible spongiform encephalopathies (TSE) are a group of neurodegenerative and infectious disorders characterized by the conversion of a normal cellular protein PrP^C^ into a pathological abnormally folded form, termed PrP^Sc^. There are neither available therapies nor diagnostic tools for an early identification of individuals affected by these diseases. New gene-based antibody strategies are emerging as valuable therapeutic tools. Among these, intrabodies are chimeric molecules composed by recombinant antibody fragments fused to intracellular trafficking sequences, aimed at inhibiting, *in vivo*, the function of specific therapeutic targets. The advantage of intrabodies is that they can be selected against a precise epitope of target proteins, including protein-protein interaction sites and cytotoxic conformers (i.e., oligomeric and fibrillar assemblies). Herein, we address and discuss *in vitro* and *in vivo* applications of intrabodies in prion diseases, focussing on their therapeutic potential.

## 1. Introduction

Prion diseases or transmissible spongiform encephalopathies (TSE) are a group of fatal neurodegenerative disorders comprising Creutzfeldt-Jakob disease (CJD), Gerstmann-Straussler-Scheinker syndrome (GSS), fatal familial insomnia (FFI) and kuru in humans, chronic wasting disease in cervids, bovine spongiform encephalopathy in cattle, and scrapie in sheep [1]. Prion diseases together with others, as Alzheimer's (AD), Parkinson's (PD), and Huntington's (HD), are also termed conformational or misfolding diseases, because they are characterized by protein misfolding and accumulation of intracellular and/or extracellular aggregates [2]. In the case of TSE, experimental evidence points to the conversion of the normal cellular prion protein (PrP^C^), into the misfolded and pathogenic form (PrP^Sc^), as the key event in the pathogenesis [3, 4]. A template-based self-propagating process underlines the generation of infectious prions. Therefore, molecules that interfere with PrP^C^/PrP^Sc^ conversion and/or neutralization serve as potential therapeutic candidates for prion diseases. Moreover, cellular cofactors, as nonproteinaceous chaperones or RNA, may play a crucial role in the generation of infectious prions and could be considered as additional therapeutic targets [5].

There are as yet no effective treatments for TSE, and immunobased approaches are emerging as important therapeutic strategies against these pathologies [6, 7]. A distinguishing feature of antibodies is that they can be specifically selected against conformational protein species including pathological conformations (misfolded monomers, oligomers, and/or fibrils), which represent major therapeutic targets of misfolding diseases. This unique property makes antibodies promising molecules against prion disease and for conformational disorders in general. Among immunotherapeutic approaches, the gene-based intracellular antibody or intrabody technology is a potentially useful platform for the treatment of neurodegenerative diseases [8]. 

This approach is based on the expression of chimeric molecules composed of specific recombinant antibodies or antibody fragments fused to intracellular trafficking sequences to be active not only in different subcellular compartments but also in the extracellular milieu [9]. This allows one to target a wide variety of neurotoxic conformers derived from misfolding-prone proteins that are generated in the cytoplasm (i.e., tau, *α*-synuclein), the nucleus (i.e., huntingtin), or the secretory compartment, inside or outside the cell (i.e., APP, *β*-amyloid peptides, prion [10–14]). Due to this definition, we consider intrabodies all recombinant antibody fragments which are engineered for a gene-based immunotherapy protocol. Many intrabodies against proteins involved in the pathogenesis of Alzheimer's, prion, Huntington's, and Parkinson's diseases have been generated and successfully applied in cellular and animal models of these diseases [8, 15]. The availability of effective *in vivo* gene delivery systems into the brain remains a major hurdle for the clinical application of intrabodies, but the growing development of new gene carriers and delivery systems holds great promise for the next future.

## 2. Intrabodies

Intrabodies are chimeric recombinant antibody fragments engineered to block or modulate the function of target proteins. The approach is a valuable tool to inhibit, *in vivo*, the function of a wide range of selected antigens both at intracellular and extracellular levels [9]. Since the first report in mammalian cells [16], intrabodies have found applications as therapeutics in infectious diseases, in cancer and in neurodegenerative disorders [8, 17, 18]. Unlike other gene-based technologies, they operate at a posttranslational level and can be selected against a precise epitope of target proteins, including protein conformers, protein-protein interaction sites, posttranslational modifications, and also nonprotein antigens.

Antibodies, as most secreted proteins, have a transient hydrophobic leader sequence which directs them through the secretory compartment. By in frame fusion with intracellular trafficking sequences, antibodies have been targeted to the cytoplasm, nucleus, endoplasmic reticulum (ER), plasma membrane [19, 20], and, more recently, also towards the degradative compartment [21]. Examples of targeting signals successfully used for intrabody application are as follows. The cytoplasmic expression of intrabodies is obtained by removal of the leader sequence of secretion (leader-less) and the nuclear targeting by adding to the leader-less antibody fragments one or more nuclear localization sequences (NLS) [22]. A signal peptide of 11 aminoacids, derived from Engrailed homeoprotein, that allows cytoplasmic single chain variable fragments (scFvs) to be secreted in the absence of classical secretion signals, has been recently identified [23]. Fusion of such a nontraditional secretion sequence to an anti-*α*-synuclein scFv enables the secretion of the intracellular target antigen [24]. Intrabody targeting to the degradative pathways is obtained by addition of a proteasomal targeting signal, such as the PEST motif of the ornithine decarboxylase [21, 25]. 

In order to prevent the appearance of receptors or resident proteins on the plasma membrane or to inhibit the secretion of selected proteins, ER-retained intrabodies are designed with a leader sequence at the N-terminus and a retention peptide, KDEL, at the C-terminus [22]. Retention in the trans-Golgi is achieved with a trans-Golgi retention signal [26]. Intrabodies targeted to the plasma membrane can be obtained by fusing a single chain antibody fragment with a receptor transmembrane domain [27].

### 2.1. Choosing an Intrabody Format

The recombinant antibody format more widely used for intrabodies is the single-chain Fv fragment (scFv) and consists of the variable domains of the immunoglobulin heavy (VH) and light (VL) chains linked with a flexible polypeptide which prevents dissociation. Other types of antibody fragments have been successfully engineered and used as intrabodies. These are the recombinant bispecific and tetravalent antibody fragments (intradiabodies) and the single domain fragments [28, 29]. The intradiabodies are made of two scFvs linked through the second and third heavy chain constant domains [30]. Single-domain fragments are composed of one variable domain (such as VL or VH chains) and are the smallest functional antibody fragments expressed as intrabodies [31, 32]. They derive from naturally occurring homodimeric heavy-chain antibodies (VHHs) present in the immune system of camelids and have excellent properties of solubility, stability and expression in mammalian cells. These molecules are easily produced, are much smaller in size, and can be engineered into new reagents with enhanced therapeutic efficacy. Due to their smaller size, they can potentially target cryptic epitopes. Recent studies have demonstrated, by intracarotid and intravenous injections into live mice, that basic VHHs (with an isoelectric point ≥9) are capable of crossing the brain blood barrier (BBB) *in vivo* and diffusing into the brain tissue [33]. Interestingly, a camelid antiprion antibody, which abrogates PrP^Sc^ replication in prion infected neuroblastoma cells, is able to transmigrate across the BBB and cross the plasma membrane of neurons, demonstrating a potential use for treatment of prion diseases [34]. Another interesting new recombinant scFv format specifically designed to cross the BBB and directed against the pathologic form of prion has been generated. In this scFv, the linker peptide was substituted with a cell-penetrating peptide (CPP) derived from penetratin. Most of the purified antiprion scFv-CPP intravenously injected was localized in brain cells, demonstrating its capacity to enter the CNS [35]. 

For clinical applications of intrabodies, generation of humanized and/or human-derived antibody domains offers obvious potential advantages. Improved strategies for *in vitro* selection of fully humanized recombinant antibodies directly from human antibody-display libraries through the creation of large natural or synthetic repertoires of antibody fragments are progressively developing [36, 37]. 

## 3. Intrabodies against Prionoses

Intrabodies can be specifically selected against conformational epitopes of amyloidogenic proteins involved in the pathogenesis of misfolding diseases. These conformational intrabodies can inhibit different stages of the aggregation process through (i) stabilization of the native state molecule, (ii) inhibition of the oligomerization process, (iii) neutralization of potentially toxic oligomeric species, (iv) inhibition of fibril formation, and (v) disruption and clearance of preformed aggregates.

It is widely accepted that the cause of prion diseases is the conformational change of the cellular prion protein, PrP^C^, from a globular to a protease-resistant *β*-sheet rich form, PrP^Sc^. Interaction between these two species raises the conversion rate and leads to generation of potentially infectious particles [4]. Therefore, blocking PrP^C^/PrP^Sc^ interaction is a major therapeutic target. Intrabodies can be used to halt this pathological interaction by different modes of action: (a) direct binding to one of the two molecular species [38, 39], (b) trapping PrP^C^ in the ER [14], and (c) rerouting PrP^C^ to the proteasome degradation pathway [40]. In particular, rerouting native proteins in precise intracellular locations is a unique property of intrabodies. In the case of misfolding prone antigens, diverting amyloidogenic plasma membrane proteins from the site of aggregation or diverting them to the degradative pathway is an attractive way to block cytotoxicity [41]. It is worth noting that the 37 kDa laminin receptor (LRP/LR) is another potential identified therapeutic target for prion diseases. Thus, LRP/LR has been shown to be a receptor of the pathogenic prion PrP^Sc^ and to play an important role in prion propagation and pathogenesis [42]. 

### 3.1. *In Vitro* Studies

Intrabody applications against prion disorders have been reported by several groups in prion-infected cell culture systems. Our group generated and stably expressed ER-retained antiprion KDEL-8H4 scFv fragments in a neuronal cell line susceptible to scrapie infection. Their intracellular expression causes a marked impairment of prion maturation and translocation towards the membrane compartment, with a strong reduction in membrane PrP^C^ levels. As a consequence, formation and accumulation of the pathogenic scrapie species, PrP^Sc^, in 139A prion strain infected cells are impaired [14]. A subsequent *in vivo* study showed that mice, intracerebrally injected with a lysate derived from KDEL-8H4 expressing cells infected with scrapie, neither developed scrapie clinical sign nor brain damage, demonstrating effective treatment [43]. The secretory version of the same intrabody (Sec-8H4), able to recognize PrP^C^ in the secretory pathway, strongly inhibits PrP^Sc^ accumulation in 139A scrapie strain infected cells. By analysing its mode of action, it was found that PrP^C^ total level is markedly reduced due to a selective rerouting of PrP^C^ to the proteasome pathway. Notably, Sec-8H4 intrabody impairs the secretion of endogenous prion molecules associated to exosomes-like vesicles, a potential spreading route for prion infectivity [40]. Drastic reduction of PrP^Sc^ accumulation was also reported by coculturing a human rhabdomyosarcoma cell line secreting antiprion scFv (6H4) fragments with chronically scrapie infected neuroblastoma cells [44]. More recently, other studies demonstrated the inhibitory effect of cell-mediated secretion of antiprion scFv fragments. The antiPrP^C^ scFvT2, derived from a new mouse monoclonal antibody which recognizes a discontinuous epitope of prion protein, was reported to inhibit scrapie accumulation by co-culturing a neuroblastoma scFvT2-secreting cell line with prion infected cells [45]. Recombinant antiprion 3S9scFv secreted by transfected HEK293T prevented prion accumulation in both 22L prion-infected N2aC24L1-3 and in Chandler prion-infected N2aC24Chm cells in a dose-dependent manner [46]. 

Another passive immunotherapy approach to treat prion disease has been attempted in cell culture by testing the monovalent version of the antiprion D18scFv through direct addition to scrapie-infected cells or by infection with lentiviral or recombinant adeno-associated viral (rAAV) vectors. Direct addition of D18scFv in scrapie-infected GT1 cells resulted in reduction of proteinase K- (PK-) resistant PrP^Sc^ level in a concentration-dependent manner. By comparing two viral transducing systems, lentiviral vectors were more efficient than rAAV in transferring of the anti-PrP D18scFv gene and in interfering with PrP^Sc^ accumulation in both ScGT1 and ScN2a cells [47]. 

### 3.2. *In Vivo* Applications

To evaluate the therapeutic effect of antiprion intrabodies *in vivo*, recombinant adeno-associated viral vectored scFvs have been applied in prophylactic TSE paradigms. rAAV is considered an ideal delivery vector in gene therapy, especially for transducing neurons in various regions of the brain [48]. The safety profiles of rAAV and its high efficiency of gene transduction have rendered this vector a valuable delivery vehicle for treating brain pathologies, including neurodegenerative disorders. Serotype 2 of rAAV (rAAV2) is one of the most commonly used vectors for brain delivery and is currently under evaluation in rAAV-based phase I/II clinical trials of Parkinson's and Alzheimer's diseases [49–51]. This vector has been employed for* in vivo* application of intrabodies in prion diseases. By using a combinatorial phagemid library of human scFvs, Wuertzer et al. identified four different PrP-specific scFv fragments, evaluated their affinity by surface plasmon resonance analysis, and assessed *in vivo* their therapeutic potential [38]. Mice were initially intracerebrally injected bilaterally into the thalami and striata with rAAV2 antiprion scFvs and, 1 month later, subjected to intraperitoneal inoculation with RML prions. Analysis of disease severity indicated that rAAV2 D18scFv, an antibody fragment specific for the putative region of PrP^C^-PrP^Sc^ interaction and with the highest affinity for PrP^C^ compared to the other scFvs, was the most effective and significantly delayed the onset of clinical signs compared to infected mice in the control group. Although all mice injected with antiprion intrabodies succumbed, rAAV2 D18scFv expressing mice demonstrated significantly extended incubation periods compared to control mice (250 days ± 8 SD versus 199 days ± 1 SD). A significant decrease of PK-resistant PrP^Sc^ burden was also observed both in white matter tracts and gray matter parenchymal regions of the brains injected with this intrabody, 27 weeks after infection, when all parameters of disease severity were assessed. Since it has to be yet established whether the accumulation of misfolded prion correlates with pathological changes and survival, as previously argued [52], the analysis of the amount of PK-resistant PrP^Sc^ at the terminal stage of the disease would be informative. Analysis of the other antiprion scFvs used in this study suggests that there is no correlation between their affinity and *in vivo* efficacy. However, the specificity of PrP^C^ epitope recognized by these scFvs should also be considered.

More recently, D18scFv was engineered into the rAAV9 vector for intracerebral injection into scrapie-infected mice [39]. rAAV9 serotype has major advantages with respect to rAAV2 including higher neuronal transduction efficiency, intracerebral diffusion, and trans-BBB neurotropism [53–55]. One month after rAAV9 D18scFv injection, mice were intraperitoneally inoculated with RML prion strain. Behavioural analysis of infected mice showed that bilateral administration of rAAV9 D18scFv into hypothalamus, thalamus, and hippocampus delays the onset of neurological symptoms compared to untreated mice (187 ± 7 days versus 166 ± 5 days). Evaluation of survival time indicated an extended time with respect to control mice, even though this result was not statistically significant. Furthermore, neuropathological assessment at early stage of disease revealed that mice expressing the antiprion scFv have lower levels of spongiosis and gliosis compared to controls, as well as PK-resistant PrP^Sc^, mostly in the thalamus, hippocampus, and caudate/putamen nuclei. This diminished level of PrP^Sc^ was also evident in mice sacrificed at the terminal stage of disease, when, differently, neuropathological changes were similar both in rAAV9 D18scFv and sham-injected mice. This result corroborates the hypothesis that severity of neuropathology and survival time are not correlated with the amount of scrapie burden, as already suggested [52]. 

As mentioned before, LRP/LR is a prion receptor and passive immunotransfer of the anti-LRP/LR scFv partly reduces scrapie burden in the spleen [56]. Microinjection of scrapie-infected mice with rAAV serotype 2 vectors encoding for anti-LRP scFv-N3 and -S18 resulted in the reduction of peripheral PrP^Sc^ propagation, without a significant prolongation of incubation times and survival [57]. It is not clear whether the difference in results between rAAV study described by Wuertzer et al. 2008 [38] and this study is due to the different choice of target (PrP^C^ versus LRP/LR) or different route of infection (intraperitoneal versus intracerebral). The fact that Zuber et al. observed reduction in PrP^Sc^ level not associated with a prolongation of incubation times and survival is a further evidence that scrapie accumulation does not automatically correlate with disease progression and infectivity. 

Another interesting study reported the generation, by infection with lentiviral vector, of a stable Ra2 microglial cell line secreting the antiprion 3S9scFv/GFP [46]. In order to evaluate the prophylactic antiprion effect of *ex vivo* gene transfer of 3S9scFv, using brain-engraftable microglial cells, Ra2 cell line was intracerebrally injected at 1 and 3 weeks before brain inoculation of mouse-adapted Chandler prion strain. Analysis of survival time showed that mice injected with 3S9scFv/GFP-Ra2 microglial cells survive longer than corresponding controls expressing GFP alone, although the effect was slight (~10 days). Assessment of PK-resistant PrP^Sc^ levels revealed no differences between antiprion scFv expressing mice and controls. In a therapeutic perspective, 3S9scFv/GFP-Ra2 microglial cells were also intracerebrally injected 7 or 13 weeks after infection with Chandler or 22L scrapie prions. In Chandler scrapie-infected mice, survival times were similar in both experimental groups, while in mice inoculated with 22L prions, there was an effect when 3S9scFv/GFP-Ra2 cells were injected 7 weeks but not 13 weeks after infection. Also in this case, the amount of PK-resistant PrP^Sc^ was not affected by the expression of antiprion scFv. It is noteworthy that authors failed to detect the injected 3S9scFv/GFP-Ra2 cells in the brain of mice at the terminal stage of disease, arguing that the low antiprion effect reported could be ascribed to a short lifetime of this cell line and/or to low scFv expression level. 

## 4. Conclusions

In the last 20 years, several antibodies have entered the biopharmaceuticals market for the treatment and diagnosis of various pathologies, including neurodegenerative diseases. Different *in vitro* studies demonstrated that antiprion antibody fragments prevent prion propagation and scrapie accumulation. Notwithstanding this fact, *in vivo* applications of these antibodies, both as passive immunization and gene-based strategy (intrabodies), failed to demonstrate a complete protection of scrapie infected animal models, even though they were able to reduce cerebral PK-resistant PrP^Sc^ and delay the onset of the disease. One concern is the choice of the therapeutic target molecule (prion monomer, oligomers, PK-sensitive or PK-resistant PrP^Sc^ or LRP/LR). So far, although different intrabodies directed to PrP^Sc^, and specific oligomeric species have been generated, the *in vivo* therapeutic efficacy still remains to be demonstrated. Another issue is the *in vivo* stability of viral vectored recombinant antibody fragments and/or the downregulation of antibody expression due to neurotoxic effect of PrP^Sc^ on antibody-expressing neurons. Both these factors could significantly decrease the therapeutic efficacy of antiprion intrabodies. 

The requirement of more effective and safety *in vivo* gene delivery systems into the brain remains a crucial issue for clinical application of intrabodies against neurodegenerative diseases. Currently, viral vectors (rAAV) are the main choice for *in vivo* delivery of intrabodies, but the growing development of synthetic gene carriers (nanomaterials) or new Trojan-horse approaches hold great promise for developing effective therapeutic antibody delivery strategies against prion diseases.
